# The influence of occupational noise exposure on blood pressure and hearing loss among female workers of childbearing age

**DOI:** 10.1186/s12889-024-19004-9

**Published:** 2024-10-01

**Authors:** Danhua Zhang, Di Wu, Sihua Wang, Jie Jiao, Yan Yang, Wenhui Zhou, Dong Zeng, Leike Li

**Affiliations:** 1 The Third people’s Hospital of Henan Province (Henan Hospital for Occupational Disease ), Henan, China; 2 Zhengzhou Occupational Disease Research Institute, Henan, China

**Keywords:** Occupational noise, Female workers, Blood pressure, Hearing loss

## Abstract

**Objectives:**

Women exposed to occupational noise experience adverse pregnancy outcomes. Therefore, we initiated a large, population-based, cross-sectional study to further investigate the effects of occupational noise on hearing and blood pressure among female workers of childbearing age.

**Study design and setting:**

A total of 6981 childbearing-aged female workers were selected for this cross-sectional study. Basic characteristics of participants were analyzed by comparing the exposed and control groups. Logistic regression models were employed to calculate the odds ratios (ORs) and 95% confidences intervals (CIs) for the associations of occupational noise with levels of hearing loss and blood pressure. The associations were further explored through stratification by age and duration of noise exposure.

**Results:**

Compared with participants not exposed to occupational noise, increasing years of occupational noise exposure were independently associated with an elevated risk of hypertension after adjustment of age, industry classification, enterprise size and economic type. Compared to participants not exposed to occupational noise, only the prevalence of bilateral hearing loss was significantly higher after adjustments for age, industry classification, enterprise size and economic type. Compared with those with normal hearing, the ORs and 95% CIs were 1.97 (0.95–4.07), 2.22 (1.05–4.68) and 1.29 (1.06–1.57) for bilateral, unilateral and any ear hearing loss, respectively.

**Conclusions:**

Occupational noise exposure is positively associated with both hypertension and bilateral hearing loss among female workers of childbearing age. Those exposed to occupational noise show an increased risk of hypertension after adjusting for potential confounders.

## Introduction

Occupational noise is the most prevalent hazard in work environments [1]. Noise acts as a stressor and is associated with multiple systemic diseases in the occupational population, such as those affecting the hearing system [2], cardiovascular system [3, 4], and endocrine system [5]. Numerous epidemiological studies have explored the associations between occupational noise exposure and conditions like hearing loss and hypertension [6, 7], with significant positive correlations identified in cohort, cross-sectional and case-control studies [8–10]. However, a lineal trend between occupational noise and hearing loss and blood pressure was significant only in male workers [11]. There is evidence that noise-induced hearing loss is affected by sex in zebrafish [12]. The work environment plays a crucial role in the physical and mental health of women. One study demonstrated that exposure to rotating shift work was significantly related to reduced childbearing and lighter birth weights in female workers [13]. Noise has been linked to menstrual cycle disorders and early miscarriage among reproductive-aged female textile workers in China [14]. A prospective cohort study in Sweden revealed that an association between occupational noise exposure during pregnancy and hearing dysfunction in children [15]. Selander et al. reported that full-time exposure to high levels of noise during pregnancy was linked with slightly reduced fetal growth [16]. In conclusion, female workers exposed to occupational noise experience adverse pregnancy outcomes. A systematic review suggested that the effects of environmental noise exposure on blood pressure and hypertension vary by sex, attributed to more than just biological differences [17]. Consequently, investigating the health impacts of occupational noise on female workers of childbearing age is essential. Moreover, the WHO/ILO review emphasizes a deficit in high-quality research on the health effects of occupational noise of women [1], highlighting an urgent public health issue concerning the health of female workers.

In summary, current research dose not fully clarify the impact of noise exposure on hearing and blood pressure in female workers of childbearing age. Therefore, we initiated a large, population-based, cross-sectional study to further investigate the effects of occupational noise on hearing and blood pressure among female workers of childbearing age.

## Methods

### Study population

A total of 6,981 childbearing-aged female workers were selected for this cross-sectional study. Women of childbearing age are defined as those aged 15–50. Recruitment occurred in 2021 through the key occupational disease monitoring information system in Henan Province, China. The study comprised 3,657 workers exposed to occupational noise as the exposed group and 3,324 subjects not exposed to noise as the control group. The noise intensity for the exposed group was characterized by exposure to a normalized continuous A-weighted sound pressure level equivalent to an 8-hour exposure of 80 dB (A). The control group was not exposed to specific noise levels in their workplaces or resident areas, complying with the hygienic standard for noise in industrial enterprises and social living environmental noise (GB3096–2008 and GB22337–2008). The control group was frequency-matched with the exposed groups based on age and residential area distribution. Workers were excluded if they lacked sufficient information regarding age, audiometric testing, blood pressure or chronic diseases such as cancer, cardiovascular disease, diabetes, liver and kidney diseases, or if they used auditory aids or had experienced drug-induced deafness. Epidemiological data were gathered through face-to-face interviews using a structured questionnaire, conducted by trained professional physicians.

### Ascertainment of hearing loss

The diagnostic criteria for hearing loss were based on the bases of the Chinese work-related health standards (GBZ49-2014, China, 2014). The audiometer tool was calibrated to determine the threshold for hearing loss. We measured air-conduction hearing thresholds at seven frequencies (0.5, 1, 2, 3,4,6, and 8 kHz) with an intensity range from − 10 to 120 dB. Pure-tone audiometry was conducted by certified audiologists in a sound-isolated room using a calibrated pure-tone audiometer. Audiometry was performed in strict accordance with the national standard (GBT16296.1-2018, China, 2018). The frequency of 1 kHz was tested twice in each ear to assess the reliability of the measurements. If the threshold difference between the two tests exceed 10 dB, the measurement was considered unreliable and excluded from the analysis. For valid measurements, the first result was used in the analysis. A hearing threshold difference between the ears of ≥ 25 dB at 0.5 and 1 kHz, or ≥ 40 dB at any higher frequencies, was also considered unreliable. If a participant did not respond at any level of a signal tone at a given frequency, a nonresponse code was recorded for that threshold, which was treated as missing data in this study. Given that hearing loss due to occupational noise exposure typically occurs at high frequencies [18], we defined bilateral high-frequency hearing loss as an indicator of chronic exposure to occupational noise. High frequencies included 3, 4, and 6 kHz. For each ear, the average high-frequency hearing threshold was calculated as the arithmetic mean of the thresholds at 3, 4, and 6 kHz [6, 11]. Hearing loss was defined as a pure-tone average of 25 dB or higher at these frequencies in any ear. Further, we categorized hearing loss into either exclusive unilateral or bilateral. Exposure duration was estimated based on years worked in noise-exposed job titles and divided into three categories: ≤1 year, ≤ 10 years, > 10 years. Participants were also grouped by age: ≤30, 30 < age ≤ 40, > 40.

### Systolic and diastolic blood pressure; hypertension definition

Following a rest period of more than 12 h after noise exposure, systolic blood pressure (SBP) and diastolic blood pressure (DBP) were measured by trained physicians using a standard protocol. Blood pressure was taken with a mercury sphygmomanometer on individuals in a seated position. SBP and DBP values were recorded as the average of three repeated measurements at 30-second intervals. Hypertension was defined as SBP ≥ 140 mmHg and/or a DBP ≥ 90 mmHg.

### Statistical analysis

Basic characteristics of participants were analyzed by comparing the exposed and control groups. Continuous variables assumed to follow a normal distribution were summarized as mean ± standard deviation (SD) and were compared using t-text. Categorical variables were presented as percentages and analyzed using Chi-square tests. Logistic regression models were employed to calculate the odds ratios (ORs) and 95% confidences intervals (CIs) for the associations of occupational noise with levels of hearing loss and blood pressure. The associations were further explored through stratification by age and duration of noise exposure. Moreover, analyses were conducted to investigate the relationship between different types of hearing loss and hypertension risk, as hearing loss may serve as an indicator of long-term noise exposure. All statistical analyses were conducted using SPSS 21.0 software (SPSS, Chicago, IL). Statistical significance was established at a *P* value of less than 0.05.

## Results

3.1 This study included 3,657 noise-exposed workers and 3,324 non-noise exposed control subjects. The basic characteristics of the participants are presented in Table 1. All subjects were female, with an average age of 36.2 years, range from 18 to 49 years. The noise-exposed female workers tended to be older and were more likely to work in large domestic manufacturing enterprises compared to the non-noise exposed group. The prevalence of hypertension was 14.4% in the exposed group and 4% in the control group (*P < 0.01*). The mean levels of SBP were 120.49 ± 16.77 mmHg in the exposed group and 117.80 ± 10.81 mmHg in the control group (*P* < 0.01). The exposed group also exhibited significantly higher levels of DBP than the control group (*P* < 0.01). Furthermore, compared to the control group, the prevalence of bilateral and any ear hearing loss in the exposed group was 15.6% and 24.2%, respectively (*P* < 0.01).

3.2 The association of occupational noise exposure with blood pressure is detailed in Table 2. Compared with participants not exposed to occupational noise, increasing years of occupational noise exposure were independently associated with an elevated risk of hypertension after adjustment of age, industry classification, enterprise size and economic type (all *P* < 0.01). Similarly, increasing years of occupational noise exposure were significantly associated with SBP and DBP, after adjustment for potential confounders (both *P* < 0.01). In subgroup analyses by age, the relationship between noise exposure duration and hypertension risk was most pronounced in the group of 30 < age ≤ 40 (all *P* < 0.01). Similarly, the DBP and noise exposure duration was significantly different in the group of 30 < age ≤ 40 (all *P* < 0.01). However, the association between SBP and the duration of noise exposure was significant in all age groups (*P* < 0.01).

3.3 The association between occupational noise exposure and hearing loss is outlined in Table 3. Compared to participants not exposed to occupational noise, only the prevalence of bilateral hearing loss was significantly higher after adjustments for age, industry classification, enterprise size and economic type (all *P* < 0.01). However, the relationship between noise exposure duration group (> 10 years) and bilateral hearing loss was not significant in the age-based subgroup analyses. Conversely, the relationship between the duration of noise exposure(≤ 1 year and ≤ 10 years) and bilateral hearing loss was most obvious in participants aged 40 or younger (all *P* < 0.05).

3.4 The association of different types of hearing loss with hypertension risk is revealed in Table 4. Compared with those with normal hearing, the ORs and 95% CIs were 1.97 (0.95–4.07), 2.22 (1.05–4.68) and 1.29 (1.06–1.57) for bilateral, unilateral and any ear hearing loss, respectively. In the age-stratified analysis, the association of bilateral hearing loss with hypertension risk was statistically significant only in participants aged 30 or younger(OR = 2.81, 95% CI = 1.49–5.29), but not in those aged over 30 (OR = 2.24, 95% CI = 0.55–7.94; OR = 1.08, 95% CI = 0.75–1.56), after adjusting for potential confounders.

**Table 1:** Basic characteristics of noise- exposed and control group

Variable	Classification	Exposed(3657)	Control(3324)	Total Population	*P*-value
Age(years), mean ± SD		38.23 ± 6.91	34.01 ± 8.49	36.22 ± 7.99	<0.01
Enterprise size, N(%)	Large	1246(34.1%)	2686(80.8%)	3932(56.3%)	<0.01
	Medium	622(17.0%)	177(5.3%)	799(11.4%)	
	Small	1214(33.2%)	381(11.5%)	1595(22.8%)	
	Micro	575(15.7%)	80(2.4%)	655(9.4%)	
Economic type, N(%)	Domestic	3345(91.5%)	1917(57.7%)	5262(75.4%)	<0.01
	Hong Kong, Macao and Taiwan investment	214(5.9%)	1278(38.4%)	1492(21.4%)	
	Foreign invested	98(2.7%)	129(3.9%)	227(3.3%)	
Industry classification, N(%)	Mining	289(7.9%)	37(1.1%)	326(4.7%)	<0.01
	Manufacturing	2457(67.2%)	2305(69.3%)	4762(68.2%)	
	Social organization	354(9.7%)	867(26.1%)	1221(17.5%)	
	Other	557(15.2%)	115(3.5%)	672(9.6%)	
Hypertension, N(%)		525(14.4%)	134(4.0%)	659(9.4%)	<0.01
SBP, mean ± SD		120.49 ± 16.77	117.80 ± 10.81	119.21 ± 14.31	<0.01
DBP, mean ± SD		77.42 ± 10.98	76.81 ± 8.31	77.13 ± 9.80	<0.01
Hearing loss, N(%)	Any ear	892(24.4%)	449(13.5%)	1341(19.2%)	<0.01
	Unilateral	320(8.8%)	259(7.8%)	579(8.3%)	0.147
	Bilateral	572(15.6%)	190(5.7%)	762(10.9%)	<0.01

**Table 2:** Association between occupational noise exposure and blood pressure

	Hypertension, *N*			SBP, *N*			DPB, *N*		
Occupational noise, exposure time (years)		Crude OR (95%CI)	Adjusted OR* (95%CI)		Crude OR (95%CI)	Adjusted OR* (95%CI)		Crude OR (95%CI)	Adjusted OR* (95%CI)
Total									
0	134/3324	1 (reference)	1 (reference)	57/3324	1 (reference)	1 (reference)	116/3324	1 (reference)	1 (reference)
≤ 1	242/1211	5.95(4.76–7.43)	4.29(3.33–5.52)	225/1211	13.08(9.70-17.64)	8.97(6.45–12.48)	188/3324	5.08(3.99–6.47)	3.64(2.76–4.80)
≤ 10	179/1540	3.13(2.48–3.95)	2.41(1.83–3.16)	130/1540	5.28(3.85–7.26)	3.98(2.78–5.70)	135/1540	2.66(2.06–3.43)	2.07(1.53–2.80)
>10	104/906	3.09(2.36–4.03)	2.94(2.12–4.07)	74/906	5.10(3.58–7.26)	4.74(3.12–7.20)	85/906	2.86(2.14–3.83)	2.80(1.96–3.99)
**Age ≤ 30**									
0	34/1149	1 (reference)	1 (reference)	15/1149	1 (reference)	1 (reference)	29/1149	1 (reference)	1 (reference)
≤ 1	44/281	6.09(3.81–9.73)	4.13(2.43–7.02)	39/281	12.18(6.61–22.46)	7.25(3.68–14.28)	33/281	5.14(3.06–8.62)	3.47(1.92–6.27)
≤ 10	21/207	3.70(2.10–6.52)	2.98(1.58–5.62)	17/207	6.76(3.32–13.77)	5.05(2.29–11.13)	10/207	1.96(0.94–4.09)	1.51(0.68–3.37)
>10	2/17	4.37(0.96–19.88)	5.01(0.97–25.80)	0/17			2/17	5.15(1.13–23.56)	5.24(0.95–28.90)
**30< Age ≤ 40**									
0	48/1262	1 (reference)	1 (reference)	25/1262	1 (reference)	1 (reference)	40/1262	1 (reference)	1 (reference)
≤ 1	121/550	7.13(5.02–10.14)	6.09(4.10–9.04)	113/550	12.80(8.19-20.00)	10.74(6.55–17.59)	94/550	6.30(4.28–9.26)	5.59(3.62–8.64)
≤ 10	90/719	3.62(2.52–5.20)	3.42(2.27–5.15)	65/719	4.92(3.07–7.88)	4.56(2.70–7.70)	65/719	3.04(2.02–4.55)	3.19(2.01–5.05)
>10	37/345	3.04(1.94–4.75)	3.55(2.08–6.05)	27/345	4.20(2.41–7.34)	4.20(2.18–8.10)	32/345	3.12(1.93–5.05)	4.23(2.36–7.57)
**Age>40**									
0	52/913	1 (reference)	1 (reference)	17/913	1 (reference)	1 (reference)	47/913	1 (reference)	1 (reference)
≤ 1	77/380	4.21(2.89–6.12)	2.75(1.78–4.27)	73/380	12.53(7.28–21.58)	7.44(4.07–13.61)	61/380	3.52(2.36–5.26)	2.27(1.42–3.62)
≤ 10	68/614	2.06(1.42-3.00)	1.41(0.89–2.24)	48/614	4.47(2.55–7.85)	2.91(1.53–5.55)	60/614	2.00(1.34-3.00)	1.38(0.85–2.25)
>10	65/544	2.25(1.53–3.29)	2.27(1.42–3.63)	47/544	4.98(2.83–8.77)	4.89(2.55–9.40)	51/544	1.91(1.26–2.88)	2.02(1.22–3.35)

*Adjusted for age, industry classification, enterprise size and economic type

**Table 3:** Association between occupational noise exposure and hearing loss

	Bilateral			Unilateral			Any ear		
Occupational noise, exposure time (years)		Crude OR (95%CI)	Adjusted OR* (95%CI)		Crude OR (95%CI)	Adjusted OR* (95%CI)		Crude OR (95%CI)	Adjusted OR* (95%CI)
0	190/3324	1 (reference)	1 (reference)	259/3324	1 (reference)	1 (reference)	449/3324	1 (reference)	1 (reference)
≤ 1	238/1211	4.04(3.29–4.95)	1.59(1.25–2.04)	128/1211	1.40(1.12–1.75)	0.88(0.68–1.13)	366/1211	2.77(2.37–3.25)	1.23(1.01–1.48)
≤ 10	254/1540	3.26(2.67–3.98)	1.59(1.25–2.03)	122/1540	1.02(0.81–1.27)	0.63(0.48–0.82)	376/1540	2.07(1.78–2.41)	1.05(0.87–1.27)
>10	80/906	1.60(1.22–2.10)	1.41(1.02–1.95)	70/906	0.99(0.75–1.30)	0.79(0.57–1.09)	150/906	1.27(1.04–1.55)	1.07(0.84–1.36)
**Age ≤ 30**									
0	25/1149	1 (reference)	1 (reference)	40/1149	1 (reference)	1 (reference)	65/1149	1 (reference)	1 (reference)
≤ 1	42/281	7.90(4.72–13.22)	3.28(1.80–5.98)	25/281	2.71(1.61–4.54)	1.83(1.00-3.34)	67/281	5.22(3.60–7.57)	2.70(1.74–4.20)
≤ 10	26/207	6.46(3.65–11.43)	4.33(2.21–8.49)	7/207	0.97(0.43–2.20)	0.73(0.30–1.77)	33/207	3.16(2.02–4.95)	2.26(1.33–3.82)
>10	0/17			2/17	3.70(0.82–16.71)	6.46(1.26–33.12)	2/17	2.22(0.50–9.93)	3.84(0.75–19.57)
**30< Age ≤ 40**									
0	68/1262	1 (reference)	1 (reference)	94/1262	1 (reference)	1 (reference)	162/1262	1 (reference)	1 (reference)
≤ 1	113/550	4.54(3.30–6.25)	1.61(1.10–2.36)	53/550	1.33(0.93–1.88)	0.84(0.56–1.27)	166/550	2.94(2.30–3.75)	1.21(0.90–1.62)
≤ 10	119/719	3.48(2.55–4.77)	1.87(1.30–2.69)	53/719	0.99(0.70–1.40)	0.74(0.50–1.10)	172/719	2.14(1.68–2.71)	1.27(0.96–1.69)
>10	14/345	0.74(0.41–1.34)	0.95(0.49–1.82)	13/345	0.49(0.27–0.88)	0.54(0.28–1.04)	27/345	0.58(0.37–0.88)	0.70(0.43–1.14)
**Age>40**									
0	97/913	1 (reference)	1 (reference)	125/913	1 (reference)	1 (reference)	222/913	1 (reference)	1 (reference)
≤ 1	83/380	2.35(1.70–3.24)	1.08(0.73–1.58)	50/380	0.96(0.67–1.36)	0.63(0.42–0.94)	133/380	1.68(1.29–2.17)	0.79(0.58–1.08)
≤ 10	109/614	1.82(1.35–2.44)	0.98(0.68–1.42)	62/614	0.71(0.51–0.98)	0.50(0.34–0.74)	171/614	1.20(0.95–1.52)	0.66(0.49–0.89)
>10	66/544	1.16(0.83–1.62)	1.11(0.75–1.66)	55/544	0.71(0.51–0.99)	0.73(0.49–1.09)	121/544	0.89(0.69–1.15)	0.89(0.65–1.21)

*Adjusted for age, industry classification, enterprise size and economic type

**Table 4:** Association between hearing loss and hypertension risk

Type of hearing loss	Hypertension, *N*	Crude OR (95%CI)	Adjusted OR* (95%CI)
No	460/5640	1 (reference)	1 (reference)
Bilateral	128/762	2.27(1.84–2.81)	1.97(0.95–4.07)
Unilateral	71/579	1.57(1.21–2.05)	2.22(1.05–4.68)
Any ear	659/6981	1.96(1.64–2.35)	1.29(1.06–1.57)
**Age ≤ 30**			
No	73/1487	1 (reference)	1 (reference)
Bilateral	22/93	6.00(3.52–10.23)	2.81(1.49–5.29)
Unilateral	6/74	1.71(0.72–4.07)	1.15(0.47–2.84)
Any ear	28/167	3.90(2.44–6.24)	2.04(1.18–3.52)
**30< Age ≤ 40**			
No	205/2349	1 (reference)	1 (reference)
Bilateral	56/314	2.27(1.64–3.13)	1.36(0.94–1.95)
Unilateral	35/213	2.06(1.39–3.04)	1.62(1.08–2.43)
Any ear	91/527	2.18(1.67–2.85)	1.46(1.08–1.98)
**Age>40**			
No	182/1804	1 (reference)	1 (reference)
Bilateral	50/355	1.46(1.04–2.04)	1.08(0.75–1.56)
Unilateral	30/292	1.02(0.68–1.53)	0.91(0.60–1.38)
Any ear	80/647	1.26(0.95–1.66)	1.00(0.75–1.36)

*Adjusted for age, industry classification, enterprise size and economic type

## Discussion

In the present study of female workers of childbearing age, we observed that occupational noise exposure is positively associated with hypertension and bilateral hearing loss. Those exposed to occupational noise exhibited an increased risk of hypertension, after adjusting for age, industry classification, enterprise size and economic type. Similarly, a two-cohort study from the United States reported that high levels of noise exposure were linked to elevated blood pressure levels [19]. Therefore, it is recommended that pregnant women avoid noisy work environments to prevent noise-induced increases in blood pressure, which may harm the mother’s health [20] and lead to adverse pregnancy outcomes [21]. In the subgroup analysis of age, we found the association between occupational noise and hypertension risk was more pronounced in the group of 30 < age ≤ 40. The potential reason is that as a risk factor of hypertension, age can partially offset the association between occupational noise exposure and hypertension. It may also be due to the gradual adaptation of the human body to noise [6]. Furthermore, we noted that occupational noise exposure in the duration group (≤ 1 year and ≤ 10 years) was associated with bilateral hearing loss risk, after adjusting for potential confounders. However, this relationship was not statistically significant among duration group (> 10 years), suggesting that bilateral hearing loss was more severe in those with short-term than long-term exposure. A similar finding was reported in a study conducted in China [22], confirming that bilateral hearing loss is a reliable biomarker reflecting cumulative personal noise exposure. Moreover, bilateral hearing loss was significantly associated with hypertension risk, particularly in those aged 30 or younger, paralleling the finding on noise exposure and hypertension risk. The relationship between bilateral hearing loss and hypertension risk could corroborate the effects of occupational noise, as hearing loss induced by occupational noise was usually supposed to be bilateral and fairly symmetrical [23]. Additionally, we also assessed the association of hypertension with unilateral hearing loss, the null association between unilateral hearing loss and hypertension also provided additional evidence for the association between occupational noise and hypertension.

Some previous studies conducted in the workplace have also explored the association between occupational noise and both hypertension and hearing loss. Kuang et al. observed that increased years of occupational noise exposure and bilateral high-frequency hearing loss were significantly associated with rises systolic and diastolic blood pressure after adjusting for age, sex, BMI, industry classification, size and economic type [11]. However, the link between occupational noise exposure time, bilateral hearing loss and hypertension risk was more pronounced in male workers [6]. Similarly, Li et al. reported that the association of occupational noise exposure and bilateral hearing loss with hypertension was significantly in males, but not in females [24]. Fredriksson et al. found a significantly increased risk of hyperacusis was found among female workers in the noise exposure group in Sweden, compared to the reference group., though hyperacusis was assessed via self-report [25]. In our study, we not only gathered a substantial cohort of female workers of childbearing age, but also assessed the degree of hearing loss using objective audiometric testing. We found a positive correlation between noise exposure and both hypertension and bilateral hearing loss. Additionally, we identified bilateral hearing loss as a sensitive marker for short-term intensive noise exposure.

### Strengths and limitations

This study has several strengths. First, it assessed the association between occupational noise and the risk of hypertension and hearing loss among a large cohort of female workers of childbearing age, using bilateral hearing loss as a confirmatory factor to enhance the validity and reliability of the findings. Second, a subgroup analysis considering by age was conducted to mitigate potential confounders. However, there are notable limitations. First, the cross-sectional design could restrict the evidence of causal inferences. Second, despite adjusting for a variety of confounders, other factors such as cigarette smoking, alcohol consumption, and psychological risk factors were not included in the multivariable analysis. Third, data on the use of hearing protection, which is crucial for assessing hearing loss, were not collected. Fourth, while we collected data on workers exposed to noise levels above 80 dB, specific noise exposure data were not obtained. In future research, we intend to collect these data to strengthen the robustness of our findings.

## Conclusions

Occupational noise exposure is positively associated with both hypertension and bilateral hearing loss among female workers of childbearing age. Those exposed to occupational noise show an increased risk of hypertension after adjusting for potential confounders.

## Data Availability

The datasets used during the current study available from the corresponding author on reasonable request.
